# Filaggrin loss-of-function variants are associated with atopic dermatitis phenotypes in a diverse, early-life prospective cohort

**DOI:** 10.1172/jci.insight.178258

**Published:** 2024-04-02

**Authors:** Samuel J. Virolainen, Latha Satish, Jocelyn M. Biagini, Hassan Chaib, Wan Chi Chang, Phillip J. Dexheimer, Michael R. Dixon, Katelyn Dunn, David Fletcher, Carmy Forney, Marissa Granitto, Matthew S. Hestand, Makenna Hurd, Kenneth Kauffman, Lucinda Lawson, Lisa J. Martin, Loren D.M. Peña, Kieran J. Phelan, Molly Shook, Matthew T. Weirauch, Gurjit K. Khurana Hershey, Leah C. Kottyan

**Affiliations:** 1 Division of Human Genetics and; 2 Center for Autoimmune Genomics and Etiology, Cincinnati Children’s Hospital, Cincinnati, Ohio, USA.; 3 Immunology Graduate Program and; 4 Department of Pediatrics, University of Cincinnati College of Medicine, Cincinnati, Ohio, USA.; 5 Division of Asthma Research, Cincinnati Children’s Hospital, Cincinnati, Ohio, USA.; 6 Pacific Biosciences, Menlo Park, California, USA.; 7 Medical Scientist Training Program, University of Cincinnati College of Medicine, Cincinnati, Ohio, USA.; 8 Divisions of Developmental Biology and Bioinformatics and Allergy and Immunology, Cincinnati Children’s Hospital, Cincinnati, Ohio, USA.

**Keywords:** Dermatology, Genetics, Allergy

## Abstract

Loss-of-function (LoF) variants in the filaggrin (*FLG*) gene are the strongest known genetic risk factor for atopic dermatitis (AD), but the impact of these variants on AD outcomes is poorly understood. We comprehensively identified genetic variants through targeted region sequencing of *FLG* in children participating in the Mechanisms of Progression of Atopic Dermatitis to Asthma in Children cohort. Twenty *FLG* LoF variants were identified, including 1 novel variant and 9 variants not previously associated with AD. *FLG* LoF variants were found in the cohort. Among these children, the presence of 1 or more *FLG* LoF variants was associated with moderate/severe AD compared with those with mild AD. Children with *FLG* LoF variants had a higher SCORing for Atopic Dermatitis (SCORAD) and higher likelihood of food allergy within the first 2.5 years of life. LoF variants were associated with higher transepidermal water loss (TEWL) in both lesional and nonlesional skin. Collectively, our study identifies established and potentially novel AD-associated *FLG* LoF variants and associates *FLG* LoF variants with higher TEWL in lesional and nonlesional skin.

## Abstract 2

Deep sequencing of the *FLG* gene in a diverse early life cohort of children with atopic dermatitis identifies variants and establishes their association with clinical and molecular phenotypes.

## Graphical abstract

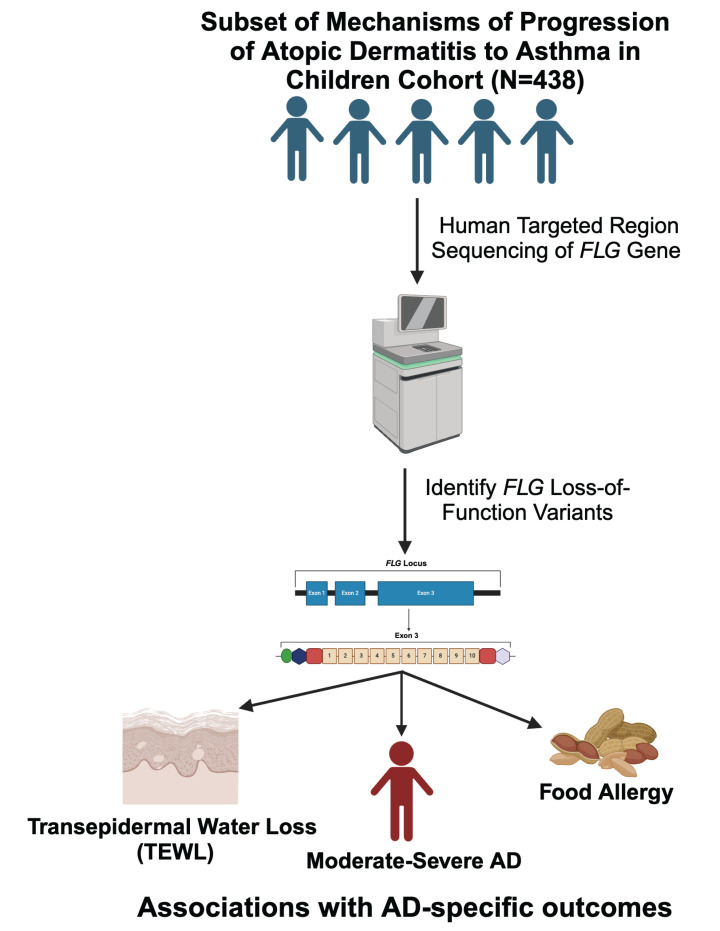

## Introduction

Atopic dermatitis (AD) is a chronic inflammatory skin disease that belongs to a spectrum of atopic disorders with shared etiology, including food allergy, allergic asthma, and allergic rhinitis. The initiation of AD is characterized primarily by a defective skin barrier that facilitates allergen entry with resulting inflammation and sensitization (1–3). AD manifests based upon a complex interaction of genetic, environmental, microbial, and immunological factors. The genetic component for AD disease risk is strong, with a 72%–86% concordance in monozygotic twin pairs and ~31 genetic risk loci robustly associated with AD, with a further ~30 recently discovered through a meta-analysis (4–6).

One of the strongest genetic predisposing factors for AD is coding mutations at the filaggrin (*FLG*) locus encoding profilaggrin, a large polyprotein that is cleaved into filaggrin monomers with important functions in terminal differentiation of the epidermis and formation of the skin barrier (7). The *FLG* gene contains 3 exons with most coding sequences localized to exon 3, a 12-kilobase segment containing 10–12 filaggrin repeats and other domains essential to filaggrin function in the skin (8). *FLG* loss-of-function (LoF) mutations, which result in nonfunctional filaggrin protein, are known to contribute to the skin barrier defect observed in many patients with AD (9). Studies estimate that 10%–40% of patients with AD have at least 1 *FLG* LoF variant, with incidence correlated with AD severity (10, 11). Lowered *FLG* expression at both the mRNA and protein levels is found in the skin of patients with AD, with low *FLG* expression in nonlesional skin likely driving severe disease outcomes (12).

The Genome Aggregation Database (gnomAD) includes 104 LoF variants in *FLG* that are categorized as pathogenic or likely pathogenic in National Center for Biotechnology (NCBI) ClinVar (13). Prior studies have identified *FLG* LoF variants in several cohorts, including R501X, S3316X, and S761fs, but the specific disease outcomes impacted by these variants are poorly understood (14, 15). The studies that originally identified *FLG* LoF variants used PCR assays specific to known *FLG* LoF variants in those of European ancestry (16, 17). Recent studies show that *FLG* LoF mutations are often ancestry specific, and thus a wider data set inclusive of diverse ancestries is necessary to more fully capture the diversity of risk variants (11, 18).

The repeating sequence of monomers in exon 3 of the *FLG* gene has historically made this region difficult to sequence (17), but recent studies have shown that deep sequencing after oligo-based DNA enrichment methods are effective and overcome this challenge (18, 19). In this study, we performed deep sequencing of the *FLG* gene in Mechanisms of Progression of Atopic Dermatitis to Asthma in Children (MPAACH), an early-life cohort of children with AD, across a range of severity, including a preponderance of mild-moderate disease scores. Prior studies have predominantly focused on cohorts of children and adults with severe AD. A majority of MPAACH children were classified as having African ancestry, an important distinction of this cohort. Our unbiased strategy allowed us to identify known novel variants in *FLG*, and we further validated this approach with long-read sequencing on those participants with more than 2 LoF variants in *FLG*. We identified the relationship of *FLG* LoF variants with clinical outcomes, such as food allergy and risk of moderate/severe AD, as well as the relationship between *FLG* LoF variants and measures of skin barrier quality.

## Results

### Unbiased deep sequencing of FLG uncovers associations between FLG LoF variants and AD.

We performed deep sequencing of the *FLG* gene in 438 individuals from MPAACH, an early-life cohort of children with AD. All children in this study had AD as defined in the Methods section. The MPAACH cohort has previously been used to define pathophysiological changes in lesional and nonlesional skin in children with AD and associate these changes with disease outcomes (12, 20). The demographics of these children are summarized in Table 1. The majority of the children in this study (69%) were of African ancestry (based on an AFR ancestry value ≥ 0.2, see Methods), with a median age equaling 1.9 years (interquartile range of 1.2–2.4). A majority (62%) of this cohort had mild AD (SCORAD < 25), and 6% had severe AD (SCORAD ≥ 50), making this cohort well suited to examine novel *FLG* variants in groups of mild, moderate, and severe AD.

**Figure 1:**
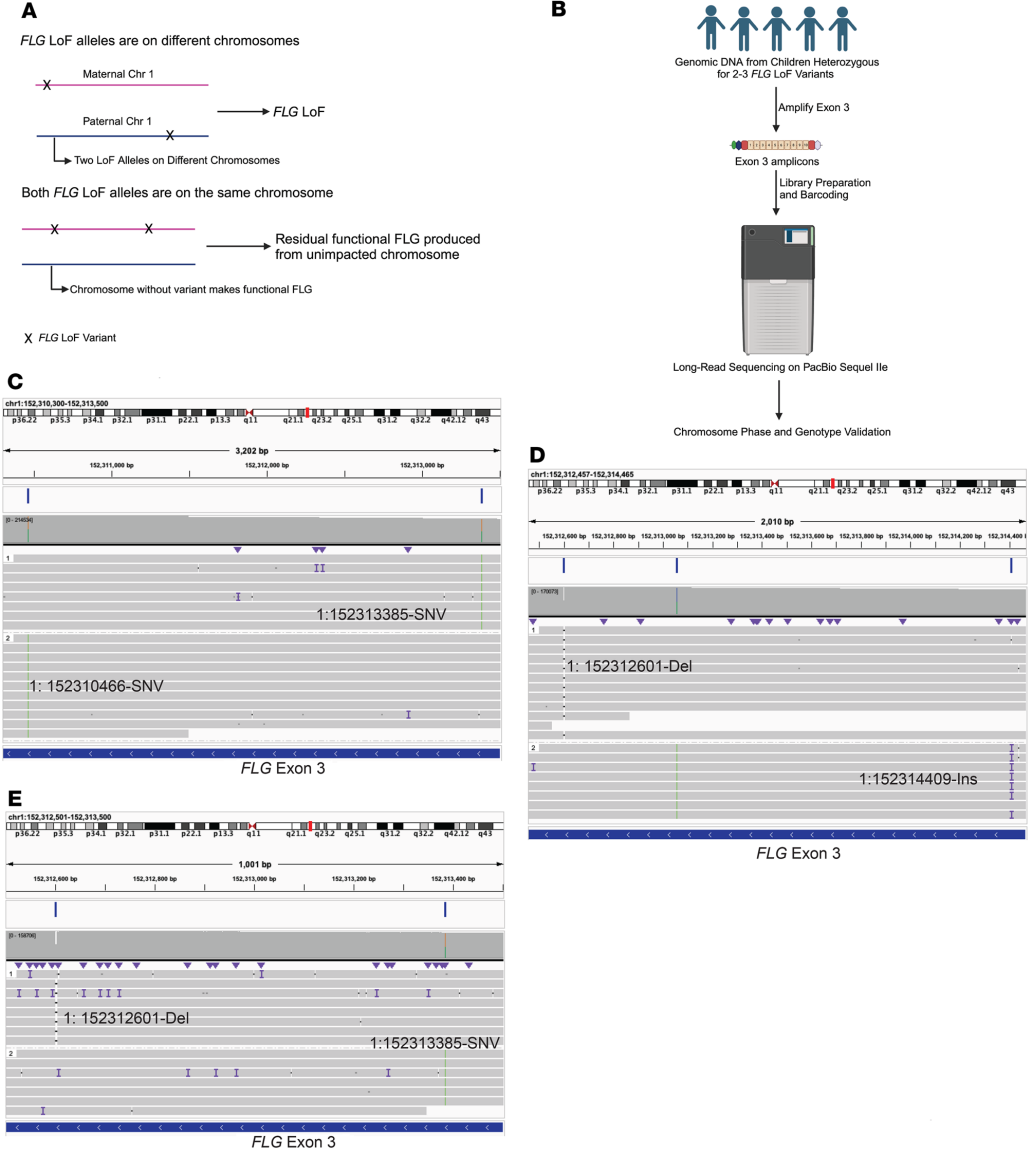
Phasing of variants to identify individuals with compound heterozygous *FLG* mutations. (**A**) Two possibilities for individuals with more than 1 LoF FLG mutation include full loss of function (top, compound heterozygote) and loss of function in 1 copy of *FLG* (bottom, with residual functional FLG produced from unimpacted chromosome). (**B**) Long-read sequencing was used to determine phase of LoF variants. (**C**–**E**) IGV visualization of BAM files from individuals 2, 4, and 5 as described in Table 2. The top portion of each screenshot shows the location of reads on chromosome 1 in hg38, with the specific *FLG* LoF variants labeled next to the corresponding mapped reads in their genomic context. Each “1” and “2” denotes different haplotypes in each individual used for phasing. *FLG*, filaggrin; LoF, loss of function; IGV, Interactive Genomics Viewer; SNV, single-nucleotide variant.

Compound LoF heterozygous mutations occur when an individual has 2 LoF variants in a gene — 1 on each chromosome. Individuals with compound heterozygous *FLG* LoF variants cannot produce any functional *FLG* (Figure 1, A and B). We initially identified 5 children in this study with 2 or more *FLG* LoF variants according to the genotype calls based upon short-read sequencing (Table 2). To determine the chromosome phase of these variants and identify those children with compound heterozygous *FLG* LoF variants, we performed long-read sequencing on DNA following PCR enrichment of exon 3 as previously published (21). The analysis of these sequences validated 3 children with compound heterozygous *FLG* LoF variants (Figure 1, C–E, and Table 2). Visualization of aligned BAM files in the IGV validated distinct haplotypes on different chromosomes for participants 2, 4, and 5 (Figure 1, C–E). Three individuals with 2 or more *FLG* LoF variants as called by short-read sequencing carried a variant identified in our short-read sequencing denoted as 1:152312606-Del, yet long-read analysis showed that this variant was an artifact, likely due to alignment errors for these individuals with 1 confirmed LoF variant. These results show that long-read sequencing is an effective method to validate short-read sequencing calls made on difficult-to-sequence regions, such as the *FLG*. The low number of participants with multiple *FLG* LoF variants precludes comparisons between these participants and those with a single LoF variant. Accordingly, this study uses a binary composite for statistical comparisons of individuals with 1 or more LoF variants compared with those with no LoF variants.

**Figure 2:**
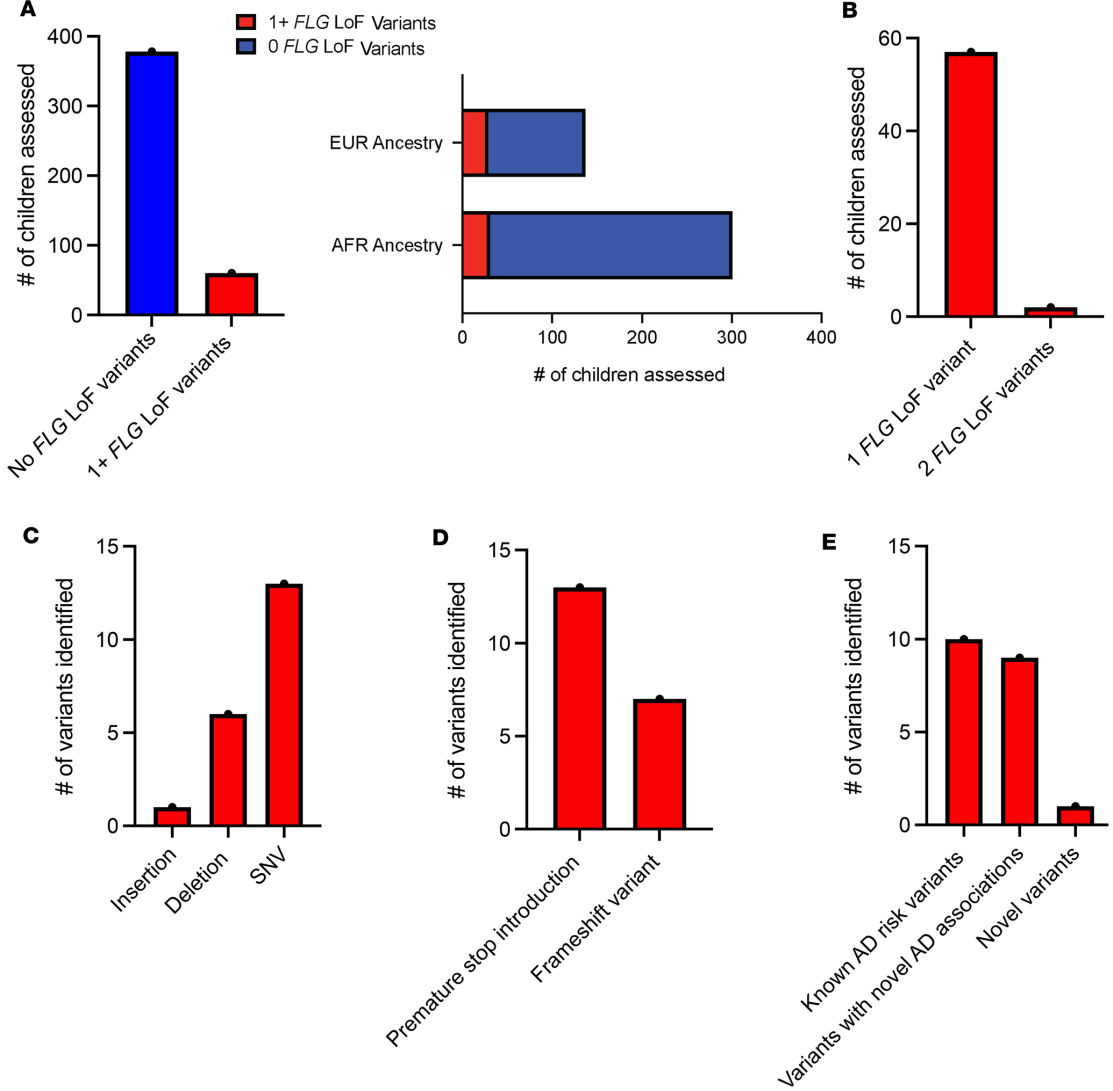
Demographics of children with *FLG* LoF variants. (**A**) The numbers of children without (blue bar) and with (red bar) LoF *FLG* variants are shown and further broken down by ancestry. (**B**) The number of participants with 1, 2, and 3 LoF *FLG* variants. (**C** and **D**) The functional annotation of identified LoF *FLG* variants. (**E**) The presence of prior association with AD and prior identification as a genetic variant. LoF, loss of function; *FLG*, filaggrin; AD, atopic dermatitis; SNV, single-nucleotide variant.

**Figure 3:**
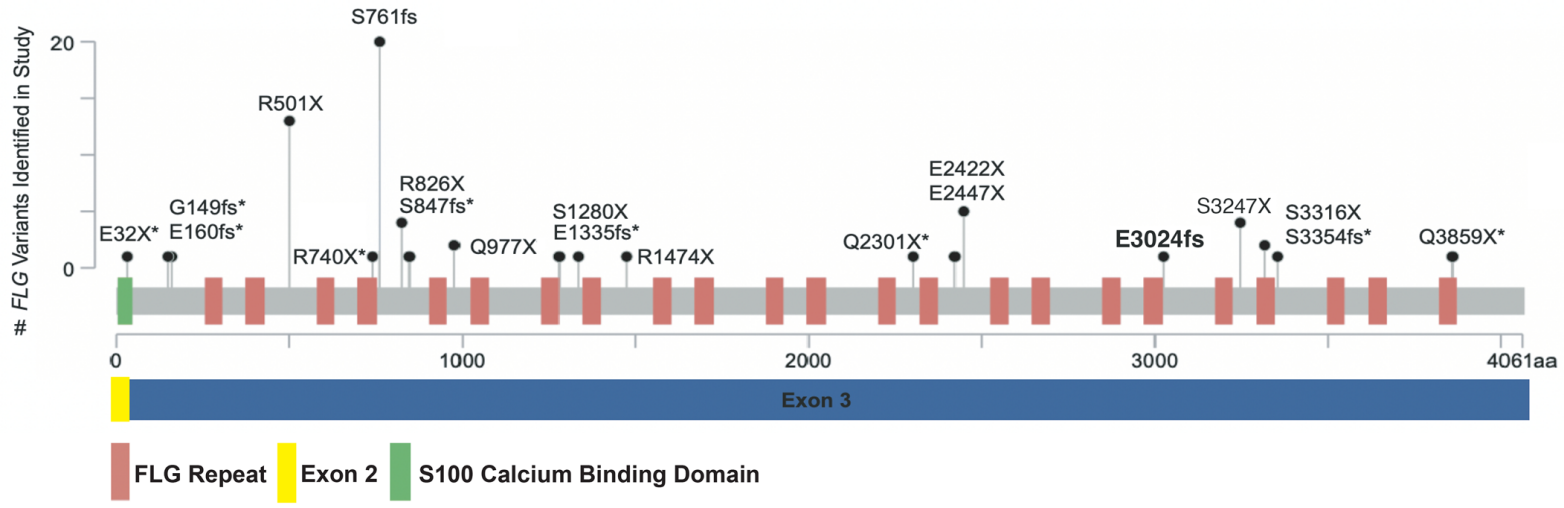
LoF *FLG* mutations identified in this study. Identified *FLG* LoF variants are presented in the context of the *FLG* genomic coordinates as a lollipop plot. The height of each variant is indicative of the number of individuals with that variant in this study. Each circle represents a variant with the amino acid change and whether the variant was previously annotated as a variant but not previously associated with AD (*) and a novel variant with no previous annotation in gnomAD or 1000 Genomes Project or dbSNP (bold). This plot was generated using the cBioPortal MutationMapper tool to reference genome GRCh38. *FLG*, filaggrin.

After quality control, alignment, variant calling, and annotation (see Methods), 20 LoF variants were identified in this cohort. Identified variants were assessed in the context of reported frequencies in gnomAD Exomes Variant Frequencies 2.0.1, BROAD, 1000 Genomes Phase 3 (1KGP), and dbSNP 155, NCBI, to assess enrichment of allele frequencies and prior discovery (Table 3). A total of 60 children (13.7%) had 1 or more of the 20 identified *FLG* LoF variants (Figure 2A), which is consistent with other studies and estimates of *FLG* LoF variant prevalence in individuals living with AD. Twenty-nine of the 60 children with 1 or more *FLG* LoF variants were of European ancestry, and 31 were of African ancestry (Figure 2A). Of the 60 children with 1 or more *FLG* LoF variants, 57 had a single *FLG* LoF variant, and 3 had 2 LoF variants (Figure 2B). The prevalence of LoF variants in European and African ancestry was 21.3% and 10.3%, respectively. The proportion of children with European ancestry who had LoF variants was significantly higher compared with those with African ancestry, with the odds of finding a LoF variant in a child of European ancestry compared with a child of African ancestry equaling 2.4 (odds ratio: 2.4, 95% CI: 1.34–4.16, *P* = 0.003). The 20 LoF variants included insertions, deletions, and single-nucleotide variants. These variants result in premature stop introductions and frameshifts that span nearly the entire length of exon 3 in the *FLG* gene and multiple repeats in the coding region (Figure 2, C and D, and Figure 3). All individuals in this study were heterozygous for the identified variants. As expected, the 2 most common LoF variants in this cohort, R501X and S761fs, are well-established *FLG* LoF mutations associated with AD. Eight additional *FLG* LoF variants identified in our study are also known AD risk variants (Table 4). Notably, 18 of the 19 of the known LoF variants identified in this study are rare, with allele frequencies less than 1% in either African or European ancestries based upon gnomAD and 1KGP. For variants identified in gnomAD and 1KGP Phase 3, we saw a high ancestry concordance in these databases with ancestry-specific variants in our study, and our study identified variants in African ancestry that are reported in gnomAD but not 1KGP Phase 3 (Table 4). A notable exception is G149fs, which has an allele frequency of 0 in gnomAD for African ancestry but is present in a participant of African ancestry in our study (Table 4). This specific participant has 99% African ancestry according to genome-wide genotyping array data (see Methods), suggesting that the low frequency of this variant may have precluded discovery of this specific variant in prior studies in African ancestry. It is also possible that the difficulty of sequencing this region has precluded prior inclusion of these variants in these databases.

Nine known variants identified in this study were present in dbSNP 155 but had no prior reported association with AD, including 4 variants that were previously identified and associated with non-AD phenotypes (Figure 2E, Table 4, and Figure 3). The variants in dbSNP are further characterized with clinical annotations in Table 4, with all variants also present in ClinGen and 16 of the variants classified as likely pathogenic or pathogenic in ClinVar for phenotypes such as ichthyosis vulgaris, AD, and a broad category of FLG-related disorders. For those variants identified in prior AD-relevant studies, a majority of the variants were also found in prior studies using the same targeted sequencing approach as our study (18, 19). We assessed the enrichment of LoF variants in our cohort compared with gnomAD and found that 11 of the 18 variants in gnomAD were enriched in our cohort (Table 4), though this enrichment was not significant after correction for multiple testing. Notably, individuals sequenced in gnomAD likely include individuals with AD due to a prevalence of approximately 20% in the general population (22), so this analysis is not a true case-control assessment.

One novel frameshift variant, E3024fs, was identified in this study, with this variant not appearing in dbSNP, gnomAD, or 1KGP Phase 3 (Figure 2E and Table 3). Positionally, E3024fs is toward the end of exon 3 and is located near AD mutations S3247X and S3316X (Figure 3). Thus, this *FLG* LoF variant is in a region of the FLG protein with well-established AD mutations and thus likely relevant to AD disease etiology. The damaging nature of the novel and known LoF mutations identified in this study is also highlighted by the low alternate allele frequencies of those variants in gnomAD and 1KGP Phase 3 and the clinical annotations from ClinVar, with the highest alternative allele frequencies occurring for those variants with established associations with AD (Table 3 and Table 4).

### FLG LoF variants are associated with higher AD severity, comorbid food allergy, and TEWL.

**Figure 4:**
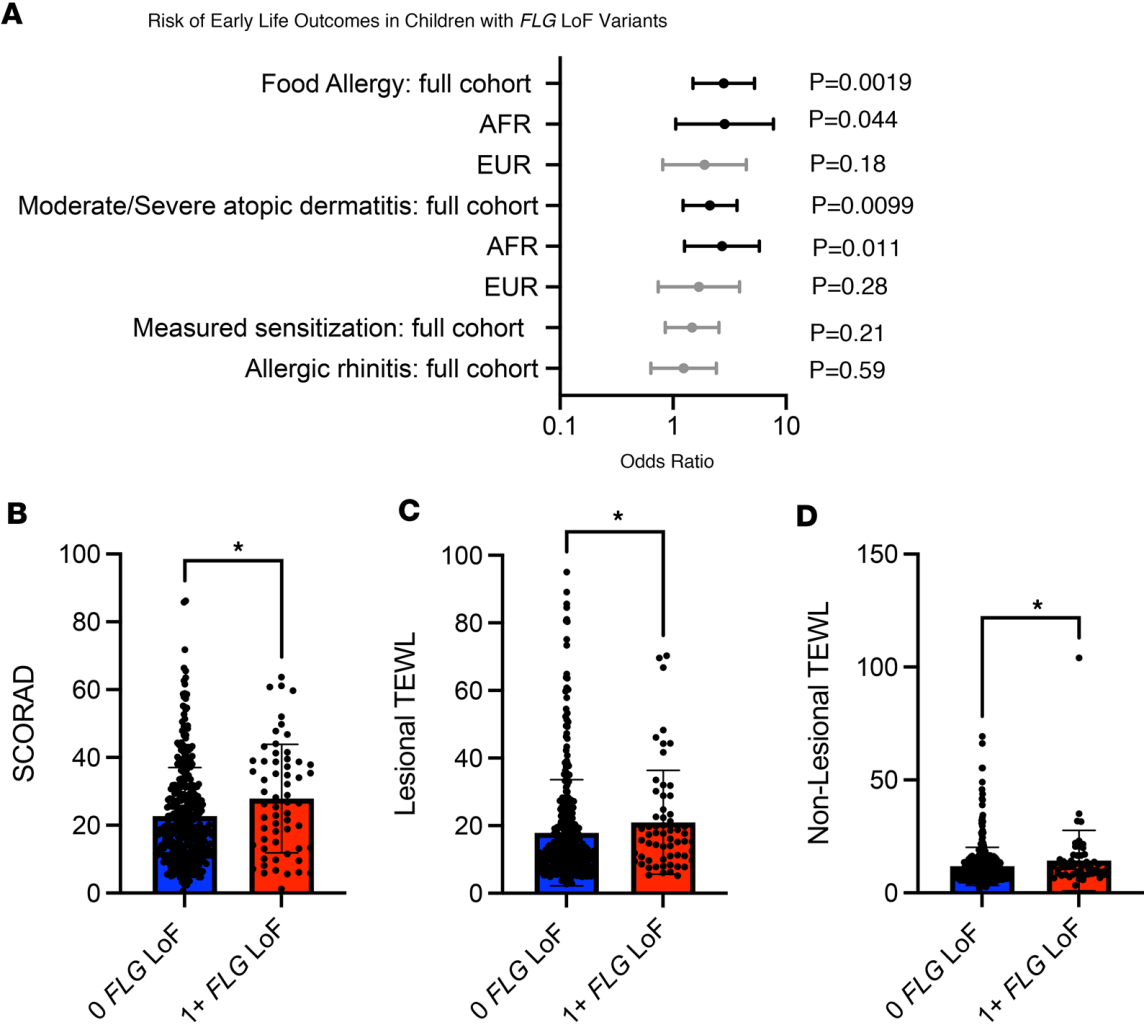
*FLG*-dependent risk of early-life allergic outcomes in the MPAACH cohort of children with AD. As described in Table 5, the association (as calculated by a Fisher’s exact test) of LoF *FLG* mutations with atopic outcomes is shown. All assessments were performed with data from the first MPAACH visit (ages 1.2–2.4 with a median age of 1.9). Outcomes are presented in the order of statistical significance in the full cohort. (**A**) Graphed odds ratios with 95% confidence intervals are provided for outcomes and annotated with *P* values (P). Ancestry-specific breakdown of association is provided for those outcomes with statistical significance in the full cohort. (**B**–**D**) The individual-level data from SCORAD, lesional TEWL, and nonlesional TEWL are presented for individuals characterized by no LoF *FLG* variants (blue) and 1 or more LoF *FLG* variants (red). Mann-Whitney assessments were used to estimate significance throughout the figure. *P < 0.05. All error bars represent ± 1 SD. AD, atopic dermatitis; TEWL, transepidermal water loss; SCORAD, SCORing for Atopic Dermatitis; *FLG*, filaggrin; AFR, African ancestry; EUR, European ancestry.

We next assessed the association between carrying 1 or more *FLG* LoF variants and AD severity. Having 1 or more *FLG* LoF variants was associated with having moderate or severe AD rather than mild disease in this cohort (odds ratio: 2.00, 1.23–3.68) (Table 5 and Figure 4A). When we assessed the association with disease severity in children of European and African ancestry separately, we identified statistically significant associations between LoF variants and dichotomous AD severity in children of African ancestry (Figure 4A). While the results did not meet the study threshold for significance, the trend of association was also found in the European subset of the cohort (Figure 4A). In a complementary analysis, we assessed the association of *FLG* LoF variants with AD severity as measured by the continuous SCORAD. Consistent with the results of the dichotomous analysis, we found a significant association between having a LoF variant and a higher SCORAD (Table 5 and Figure 4B). The SCORAD association demonstrated similar trends when stratified by ancestry but did not meet study thresholds for significance in children of European ancestry (Supplemental Figure 1, A and B; supplemental material available online with this article; https://doi.org/10.1172/jci.insight.178258DS1). This is likely due to reduced statistical power because of a lower number of children of European ancestry in this study as presented in Supplemental Table 1.

Having 1 or more *FLG* LoF variants in this cohort was also associated with a higher risk of parental report of physician-diagnosed food allergy with an odds ratio 2.81 (95% CI: 1.50–5.26) (Table 5 and Figure 4A). This is consistent with prior studies showing that *FLG* LoF mutations are associated with food allergy even when controlling for coexistent AD (23, 24). Having one or more *FLG* LoF variants was not associated with broad sensitization to any allergens, allergic rhinitis, or the PARS (see Methods for outcome definitions) in this study (Table 5, Figure 4A, and Supplemental Figure 3).

In addition to AD-associated clinical outcomes, we assessed the relationship of *FLG* LoF variants with functional measurements taken in the skin of MPAACH children. TEWL is a measure of the amount of water that escapes from the stratum corneum per area of skin, thereby quantifying skin barrier integrity (25). TEWL was measured in both lesional and nonlesional skin of the children in this cohort. Having 1 or more LoF variants was associated with a higher TEWL in both lesional and nonlesional skin (Table 5 and Figure 4, C and D). In an analysis stratified by ancestry, the association trends are replicated, with the *P* value not meeting study thresholds for significance (Supplemental Figure 1, C–F). Taken together, the association of *FLG* LoF variants with skin barrier function, AD severity, and food allergy suggest that *FLG* LoF variants can play a role in driving more severe disease in children with these variants, a potentially novel finding in the context of an early-life cohort of children with a majority having mild disease.

### LoF mutations at the beginning of the FLG protein are associated with FLG mRNA expression in nonlesional skin.

**Figure 5:**
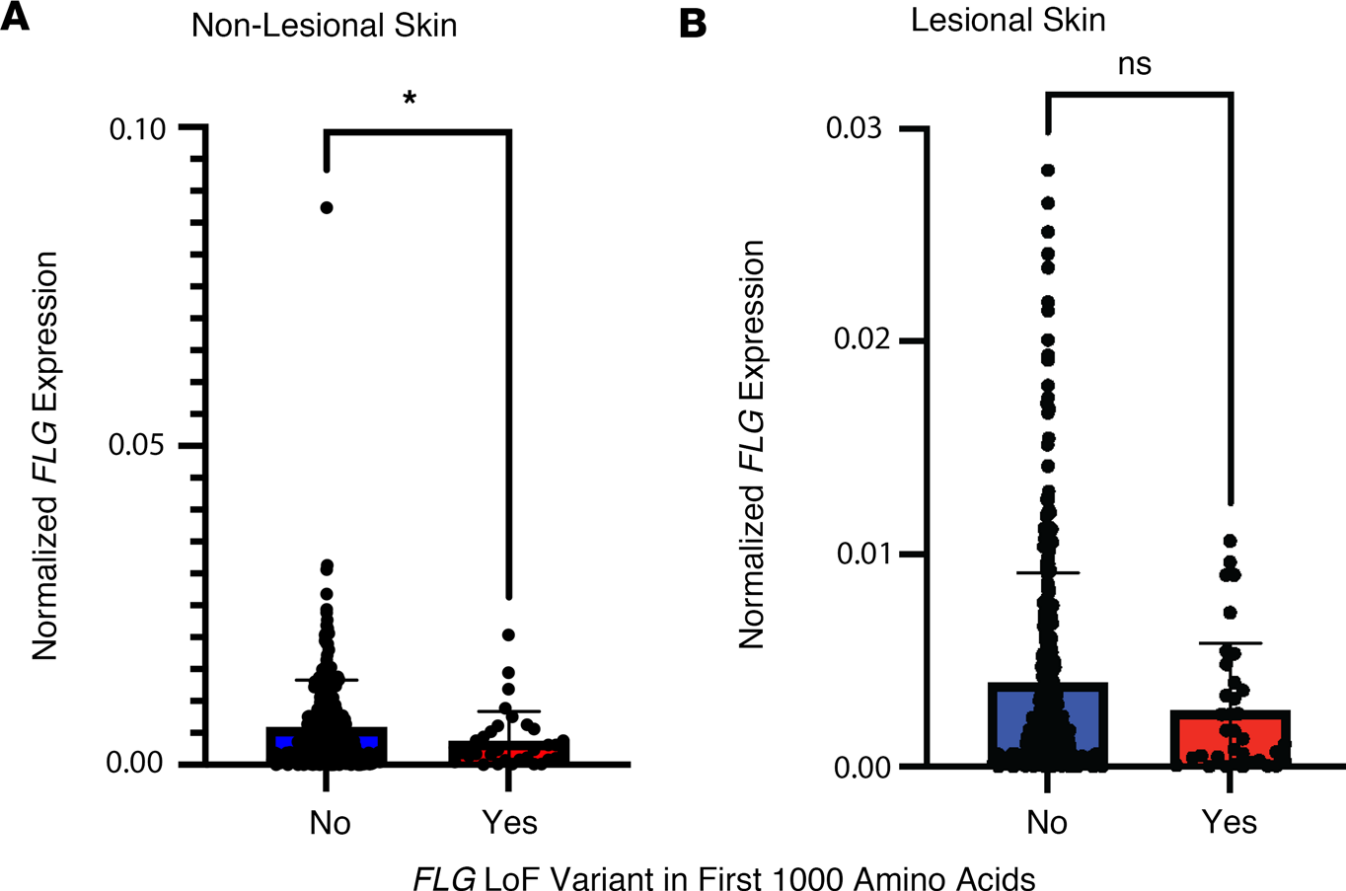
Having an *FLG* LoF variant in the first 1,000 amino acids of the FLG protein is associated with lower *FLG* expression in nonlesional skin of children with AD. Individual-level data of *FLG* mRNA expression values in nonlesional (**A**) and lesional skin (**B**). Mann-Whitney assessments were used to estimate significance. *P < 0.05. All error bars represent ± 1 SD.

Low *FLG* expression has been associated with development of moderate to severe AD, particularly in nonlesional skin (12, 26). We thus assessed whether having any *FLG* LoF variant was associated with low *FLG* mRNA expression in both lesional and nonlesional skin of MPAACH children. Having 1 or more *FLG* LoF variants was not associated with low *FLG* mRNA expression in the full cohort or when stratifying by ancestry (Supplemental Figure 2). LoF variants in general can lead to a reduction in mRNA for the respective protein. For example, some LoF variants can result in reduced mRNA through nonsense mediated decay (NMD) mechanisms, with LoF mutations that occur earlier in the coding region being more likely to trigger NMD (27, 28). Thus, to test whether LoF variants at the beginning of the FLG protein are associated with reduced *FLG* mRNA, we adjusted the genetic composite to only include variants in the first 1,000 amino acids of the protein. This included the following variants: E32X, G149fs, E160fs, R501X, R740X, S761fs, R826X, S847X, and Q977X. Notably, this composite included R501X and S761fs, which were the most common variants in our study and are well-established AD risk variants. In the refined analysis, we found a significant association between *FLG* LoF variants and *FLG* mRNA expression in nonlesional skin (Figure 5A). This nonlesional skin–specific finding is notable in the context of prior studies showing that low *FLG* expression is associated with AD and particularly moderate to severe AD in nonlesional skin (12). While the trend was evident, the association of early-transcript *FLG* LoF variants and *FLG* mRNA expression did not meet the study threshold for significance in lesional skin (Figure 5B). Of the children with low nonlesional *FLG* mRNA (defined as relative expression in the bottom 25%), 15.3% of these children had an *FLG* LoF variant.

Both early-gene *FLG* LoF variants and AD severity are associated with *FLG* mRNA (Figure 5) (26). We were unable to decouple these overlapping associations, as we could account for the association of having 1 or more *FLG* LoF variants in the first 1,000 amino acids with *FLG* expression by including SCORAD in a logistic regression model and account for the association of SCORAD with *FLG* expression by including the composite score representing the presence of 1 or more *FLG* LoF variants in the first 1,000 amino acids as a covariate in a logistic regression model.

## Discussion

In this study, we show that *FLG* LoF variants in MPAACH children (with primarily mild-moderate AD) are associated with increased risk for AD-associated outcomes and lower skin barrier integrity. We further demonstrate the association of early-peptide *FLG* LoF variants with reduced *FLG* expression. This study builds upon prior studies showing that targeted region sequencing is an effective method to overcome sequencing challenges associated with repeats in the *FLG* gene (19). In our study, this targeted region sequencing identified both known and novel variants in children of European and African ancestry (18). We further show that a complementary approach such as long-read sequencing may be necessary when confirming novel variants in this genomic region.

To our knowledge, this study is the first to apply this technology and assess the impact of the sequenced *FLG* LoF variants on clinical outcomes and disease-associated measurements. Further, as *FLG* variants vary significantly by race and ancestry (18) and few previous studies have included cohorts of African ancestry, our study fills a significant gap finding *FLG* LoF variants in this population. Indeed, Margolis et al. established that studies focused on a small subset of *FLG* LoF variants leave individuals of African ancestry understudied because variants specific to African ancestry are not fully known (18). Our study cohort highlights that *FLG* LoF variants have not been comprehensively identified in African ancestry, as we identified 20 *FLG* LoF variants spanning the length of *FLG* exon 3 with 4 variants (R2447X, Q977X, S761fs, and R501X) shared in European and African ancestry, 10 variants found exclusively in African ancestry (Q3859X, S3354fs, S3316X, E3024fs, Q2301X, S847X, R826X, R740X, G149fs, and E32X), and 6 (S3247X, E2422X, R1474X, E1335fs, S1280X, and E160fs) found in European ancestry. When comparing the frequencies of known variants in this study to frequencies in gnomAD and 1KGP, we see high ancestry concordance between our cohort and these databases.

While the established AD risk S761fs LoF *FLG* mutation has an allele frequency of 1%, the vast majority of previously identified *FLG* LoF variants were quite rare (gnomAD and 1KGP minor alleles frequencies as low as 0.0008%). Four *FLG* LoF variants were identified in this study that had not previously been associated with AD in LitVar, yet these variants were classified as either likely pathogenic or pathogenic in ClinVar. Our study thus increases confidence in these ClinVar classifications. One novel *FLG* LoF variant was identified in this study in a individual of African ancestry (E3024fs). This novel variant was not previously identified in gnomAD or 1KGP, likely because of the extremely rare allele frequencies in non-African ancestry populations. The known and novel *FLG* LoF variants identified in this study are located near established damaging variants, increasing confidence that they may be important in the etiology and pathogenesis of AD.

Our study also incorporates both lesional and nonlesional skin paradigms into a study of *FLG* LoF variants, an additional novelty in our approach. Prior studies have shown that events in the nonlesional skin are critical to the inflammatory feedback loop and events that lead to the development of AD (12). Our association of *FLG* LoF variants and food allergy (FA) affirms findings in prior studies finding that these variants increase risk for risk of FA independent of AD status, providing mechanistic insight into the atopic march from skin barrier defects to AD and subsequent FA (23, 24). We further show that a specific subset of *FLG* LoF variants is associated with lower *FLG* expression in nonlesional skin, a critical event to subsequent lesional skin development (12). We also find reduced expression of *FLG* in the nonlesional skin of children with *FLG* LoF variants in the first 1,000 amino acids of the protein, which suggests a secondary mechanism of NMD. It is challenging to develop genetic tests for *FLG* mutations since children are likely to have clinical manifestations by the time they receive results of a genetic test. Our study shows that genetic risk for AD may manifest through decreasing *FLG* expression in predisease nonlesional skin, a finding that could inform diagnostics and treatments for AD.

Strengths of this study include the focus on an early-life cohort of children with relatively mild disease. The cohort’s predominance of children of African ancestry also allowed us to identify *FLG* LoF mutations in a historically understudied group. This study also included several limitations. While the current gold standard for FA diagnosis is oral food challenge, FA in MPAACH was defined as parental report of physician diagnosis. While parent-reported FA can have the potential for false reporting and include food intolerance, we observed the parent report of physician-diagnosed FA to be 15% of the cohort. This proportion of FA diagnoses is consistent with expectations given that about a third of children with moderate-severe AD have comorbid FA (29). The SCORAD assessment at visit 1 reflects the AD severity at enrollment. While participants are asked to discontinue topical treatments, it is possible that some measurements could be impacted by use of topical creams and medications in those with more severe disease. Finally, we were unable to assess the individual contributions of AD severity (as measured by SCORAD) and early transcript *FLG* LoF variants on *FLG* expression due to the correlation of these variables (30–32).

Collectively, our study identifies established and new AD-associated *FLG* LoF variants across a diverse childhood AD cohort with a range of disease severity. We further identified association of these LoF variants with clinical AD outcomes and functional skin barrier disruption in lesional and nonlesional skin of children with AD. Understanding the role of *FLG* LoF variants in patient outcomes has important clinical implications. *FLG*-based treatments have been widely proposed as viable therapeutics for AD (33, 34). Some of these treatments involve direct supplementation of *FLG*, while others involve indirect supplementation of *FLG*-related metabolites. The findings from this study expand the number of potentially actionable *FLG* LoF variants associated with AD in children of both European and African ancestral groups.

## Methods

### Sex as a biological variable.

Our study cohort included male and female participants, and findings are reported without regard to sex.

### Study individuals.

MPAACH, the first US prospective longitudinal early-life cohort of children with AD who complete annual visits with extensive biospecimen collection, has been previously described (12). Recruitment began in December of 2016 and is ongoing. Briefly, children in the greater Cincinnati area (southwest Ohio and northern Kentucky) were identified through public advertising or the medical chart at Cincinnati Children’s Hospital Medical Center (CCHMC). The recruitment details, exclusion and inclusion criteria, and biospecimen collection and processing of the MPAACH cohort have been detailed previously (12). Briefly, eligible children were aged ≤ 2 years at enrollment, were born after a gestation ≥ 36 weeks, and either had a diagnosis of AD based on the Hanifin and Rajka Criteria for Atopic Dermatitis (35) or had parent/legal authorized representative indication of a positive response to each of the 3 questions from the Children’s Eczema Questionnaire (36, 37). Exclusions include a comorbid lung condition, dependence on immunosuppression or oral steroids for a medical condition other than asthma, conditions precluding biological sample collection or spirometry, and a bleeding diathesis.

This study was approved by the institutional review board at CCHMC under protocol number 2016–5842, and all individuals provided informed consent/parental permission before participation. For the present study, blood collected from 452 participants was subjected to targeted capture sequencing of the *FLG* gene.

### Demographics, race, and ancestry.

Participants provided a self-reported race upon enrollment in the study cohort. Additionally, genome-wide genotyping arrays were used to impute genetic ancestry. The methods used to determine ancestry in this cohort have been previously published (20). Briefly, we used 592 ancestry informative markers from the MEGA chip to infer population structure using the Structure software package (Pritchard Lab, Stanford University). We assumed an admixture model and independent allele frequencies, with a burn-in period of 5,000 and 10,000 MCMC reps after burn-in. When running structure simulations, we set the number of populations assumed to 2. A threshold ≥ 20% African ancestry from the array results was used to classify individuals in this study as being of AFR ancestry, and individuals below this threshold were classified as being of European ancestry. Seven individuals were missing genome-wide genotyping array data, and for these individuals self-reported race was used (“Black” self-identification corresponding to African ancestry and “White” self-identification corresponding to European ancestry). Prior studies show a very strong concordance of self-reported race and ancestry in this cohort (20).

### Outcome definitions.

All outcomes were assessed at visit 1, when the child was enrolled into the MPAACH cohort (age 0–2 years). A positive skin prick test (SPT) is defined as a wheal measurement ≥ 3 mm larger than the saline control to a food or aeroallergen. AD severity was assessed by SCORAD utilizing the SCORAD app by study staff trained by a study physician: mild AD severity was characterized as a SCORAD < 25, moderate as SCORAD ≥ 25 and < 50, and severe as SCORAD ≥ 50. Food allergy was defined as parental report of a physician diagnosis of FA. Allergic rhinitis was defined as parental report of ‘‘a problem with sneezing or a runny or a blocked nose when he/she DID NOT have a cold or the flu,’’ and/or of 1 or more allergy symptoms (itchy red eyes, runny nose, sneezing, itchy nose, congestion) and/or report of the child scratching or itching his/her eyes when he/she is in the same room with a cat, a dog, disturbance of dust, or is near freshly cut grass, and has had at least 1 positive SPT result to an aeroallergen. The PARS was calculated as published (38). High asthma risk was defined as a PARS score ≥ 9. The respiratory symptom burden was defined according to our validated and published respiratory symptom score (maximum weekly symptom frequency of wheeze, cough, chest tightness, or shortness of breath). TEWL was determined at both lesional and nonlesional sites at each visit using the DermaLab TEWL probe by clinical research staff who have been trained by the study physicians (Cortex Technology).

### DNA isolation.

DNA was extracted from MPAACH participants’ whole blood, granulocyte blood, or saliva. Total blood was collected using EDTA or sodium heparin tubes, and PBMCs collected from sodium heparin tubes were purified using Ficoll. Saliva was collected using the OG-675 kit per manufacturer’s instructions (DNA Genotek). DNeasy Blood and Tissue Kit (QIAGEN) was used to extract DNA from whole blood, granulocyte blood, and PBMCs. Saliva DNA was extracted using the prepIT-L2P kit (DNA Genotek). DNA was extracted using the kits mentioned above, either manually or using a QiaCube Connect (QIAGEN) machine. DNA was eluted in Buffer AD (10 mM Tris-HCl, 0.5 mM EDTA, pH 9.0).

### RNA isolation and quantitative PCR.

Skin tape strips were collected from nonlesional or lesional skin at each MPAACH visit as described and validated previously (12, 39). As previously described, lesional skin was defined by the Hanifin and Rajka diagnostic criteria for AD (35). Nonlesional skin was defined as at least 10 cm from current or historical lesional sites and no history of lesions at that site according to parental report. A total of 12 tape strips for each sampled skin site were collected at each visit. RNA was extracted from tapes 8 and 9 using Promega ReliaPrep RNA Miniprep System. RNA was reverse-transcribed using SuperScript IV VILO Master Mix. qPCR was performed on a QuantStudio 3 Real-Time PCR System (Thermo Fisher Scientific) using TaqMan gene expression assays for *FLG* (Hs00856927_g1) and *18S* as housekeeping control (Hs03003631_g1) and TaqMan Fast Advanced Master Mix (Thermo Fisher Scientific) with appropriate controls as validated previously (12, 39). *FLG* expression levels were normalized to *18S* levels using the method (40). The maximum gene expression value from either tape strip 8 or 9 was selected to represent *FLG* expression for each individual.

### Deep (target capture) sequencing of the FLG gene.

Following extraction, DNA was prepped for deep sequencing and hybridized with Agilent SureSelect probes designed to cover 100% of exons and UTRs in the *FLG* gene following the strategy outlined in prior studies (18, 19). Extracted DNA was checked for quantity and quality using a Qubit3 fluorometer (Thermo Fisher Scientific) and the Agilent TapeStation 4150 System, respectively. DNA integrity number (DIN) values for the DNA samples were 8.00–9.00. A higher DIN value indicates a higher degree of integrity. The samples that passed the quality control in our laboratory were submitted to Novogene for deep sequencing of *FLG*, and a second quality check was run by Novogene prior to subjecting the samples to sequencing. The probe design was based on the reference genome hg38. The total target region size is 12.853 kbp, and the total probe size is 13.080 kbp, with the expectation that the probe can cover 100% of target regions. Sequencing was performed on an Illumina NovaSeq 6000, yielding paired end reads of 150 bp.

### Genetic analysis.

The resulting sequencing depth following enrichment and deep sequencing was over 1,000 times per base. Sequencing data were aligned to GRCh38 using the Burrows-Wheeler Aligner (41) program, and single-nucleotide variants and insertion/deletions were called by Genome Analysis Toolkit (GATK) HaplotypeCaller (42) version 4.2.0.0 following best practices. A set of hard filters was applied based on GATK best practices, with filters for strand bias, quality by depth, mapping quality, read depth, and allelic depths. These filters resulted in a high-quality set of variants with an average variant call rate of 0.98 and an average sample call rate of 0.98. Variants meeting study quality thresholds were annotated using RefSeq Genes 109.20200815 v1, NCBI, to classify variants as LoF, missense, or UTR variants. The LoF variants identified in this study were of especially high quality, with an average call rate of 0.99 (range 0.86–1) per LoF variant. Filtered variants were annotated and compared with variants curated in gnomAD Exomes Variant Frequencies 2.0.1, BROAD, 1000 Genomes Phase 3 (1KGP Phase 3), dbSNP 155 NCBI, and ClinVar 2023-04-06 v2 NCBI.

### Long-read sequencing and exon 3 enrichment for potential compound heterozygotes.

Five individuals (out of 438) in this study were determined to have 2 or more heterozygous *FLG* LoF variants via short-read sequencing. To validate phase and genotype for these 5 samples, DNA from blood was extracted as described above, and a published protocol was used to PCR-amplify exon 3 for long-read sequencing (21). Briefly, the first PCR reaction used forward: 5′TGGGACAGTGATTATGTTGGAGA3′ and reverse: 5′AGGGTTATTTTGAGCTCTTTGTGAA3′ primers to amplify a 12-kilobase region that was then amplified in a second nested PCR reaction with forward primer: 5′GGCGCAGACTGTCCATGGGT3′ and reverse primer: 5′TCGGCAAATCCTGAAGGTAAGAGTC3′. Starting with 300 ng of input DNA, samples were prepped using a SMRTBell Prep Kit 3.0 (catalog 102-141-700) and Sequel II Binding Kit 3.2 (catalog 102-194-100). Barcodes used were from SMRTBell Barcoded Adapter Plate 3.0 (catalog 102-009-200), and sequencing and demultiplexing were performed on a PacBio Sequel IIe with sequencing kit 2.0 (catalog 101-820-200) and 30-hour movies on an 8M SMRT Cell. Reads were aligned to hg38 and variants called and phased using the “Variant Calling” application in SMRT Link v13.0.0.

### Statistics.

All variant annotation, analysis, and composite construction was conducted in Golden Helix SNP & Variation Suite (SVS) version 8.9.1. Statistical analyses were performed in GraphPad Prism 9 and Golden Helix SVS. Using a standard analytical approach (18), associations were assessed based upon an *FLG* LoF composite (i.e., presence of 1 or more *FLG* LoF variants or no *FLG* LoF variant). Before assessing statistical association with disease outcomes, a power calculation confirmed 66–87% statistical power to detect disease associations with alpha equaling 0.05 (Supplemental Table 1). Given this moderate statistical power to identify associations, we choose an analytical strategy that focused on full cohort associations (Supplemental Table 1). We followed these analyses with less robustly powered ancestry-specific analyses that allowed us to refine our understanding of ancestry for associations that were significant in the full cohort. For dichotomous outcomes, the Fisher’s exact test was used, while continuous variables utilized a Mann-Whitney test, as data sets were determined not to follow a normal distribution (Supplemental Table 1). Two-tailed tests were used in all statistical analyses. Correction for multiple testing was performed using a Holm-Šidák correction for atopic outcomes and a Bonferroni correction for enrichment of LoF variants in gnomAD. For all statistical tests, *P* < 0.05 was considered statistically significant.

### Study approval.

This study was approved by the institutional review board at CCHMC under protocol number 2016–5842, and all individuals provided written informed consent/parental permission before participation.

### Data availability.

Source data for this manuscript are deposited in dbGaP under identifier phs003489.v1.p1. Supporting Data Values are available for figures in the Excel file.

## Author contributions

LJM, GKKH, and LCK developed the study design. SJV led the analysis, and LS, LJM, GKKH, and LCK supervised the study. LS, JMB, WCC, and KJP were responsible for cohort stewardship and database management. SJV, LS, HC, MRD, DF, KD, MH, CF, LL, and MS offered experimental support. SJV, PJD, MG, KK, LL, LJM, MSH, LDMP, KJP, and MTW offered analytical support. All authors read and edited the manuscript.

## Supplementary Material

Supplemental data

Supporting data values

## Figures and Tables

**Table 1:**
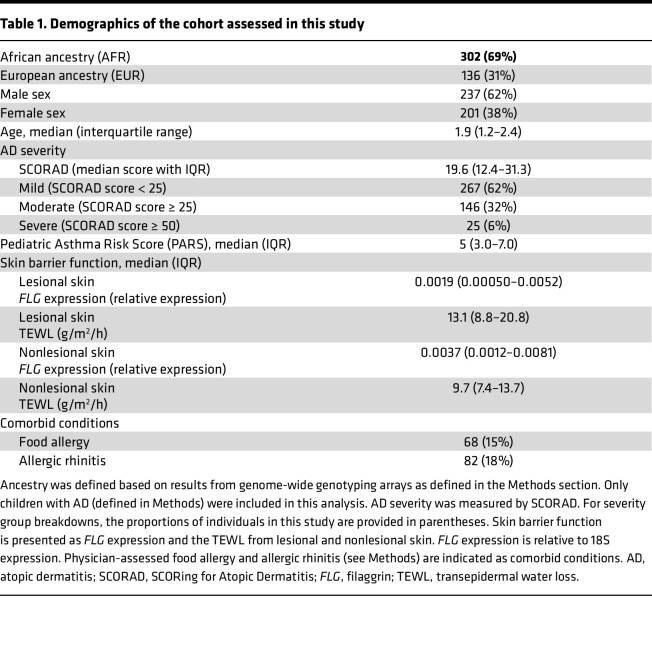
Demographics of the cohort assessed in this study

**Table 2:**
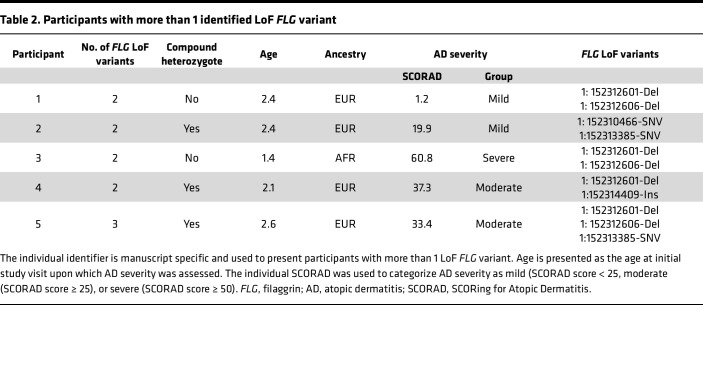
Participants with more than 1 identified LoF *FLG* variant

**Table 3:**
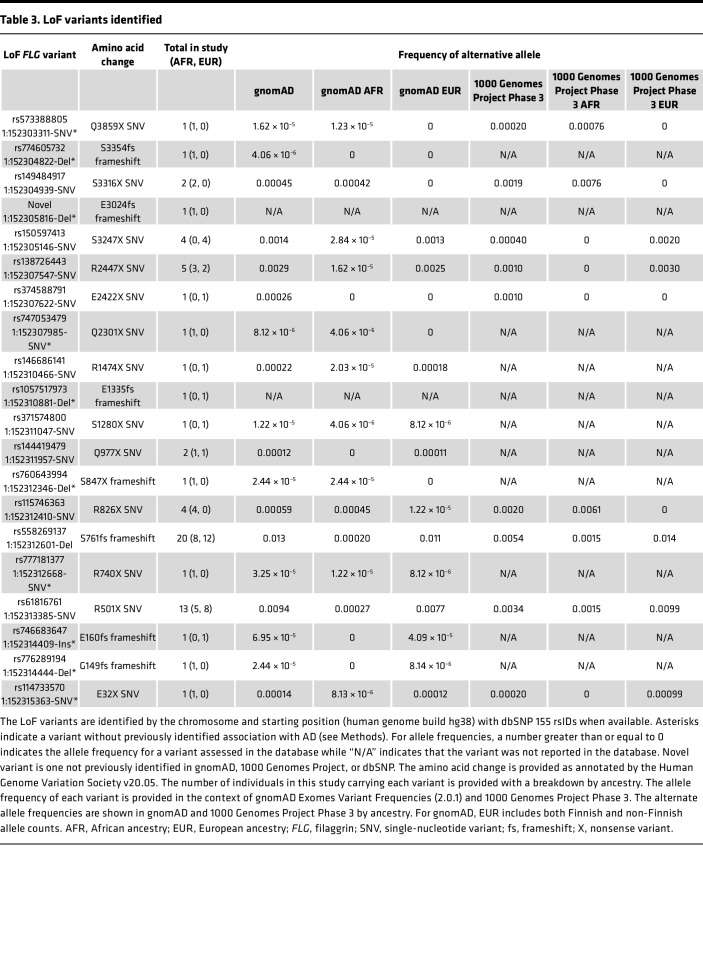
LoF variants identified

**Table 4:**
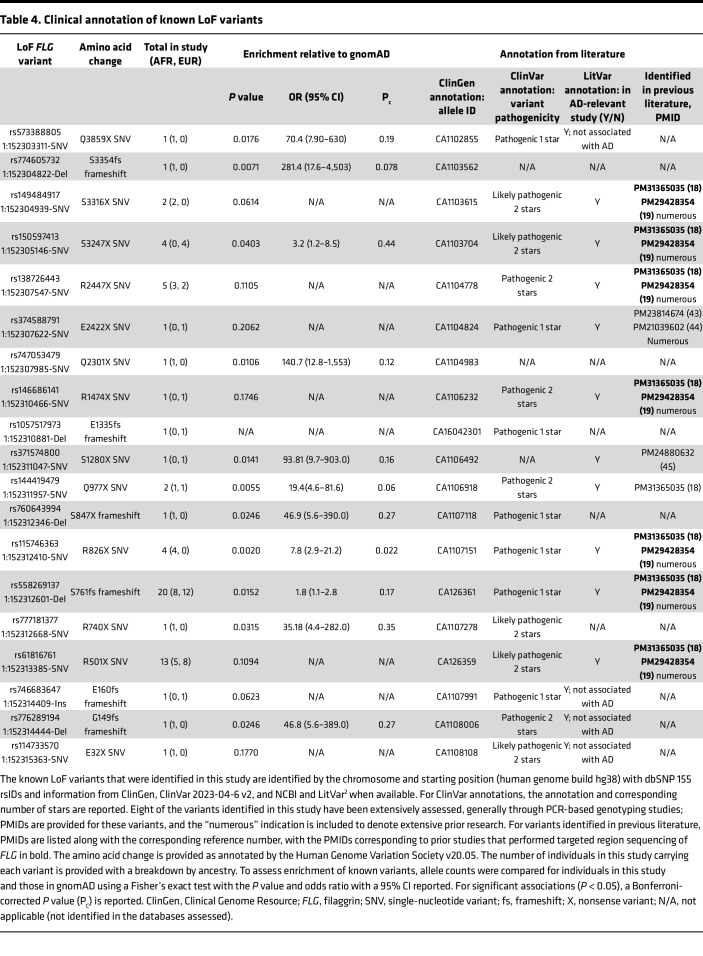
Clinical annotation of known LoF variants

**Table 5:**
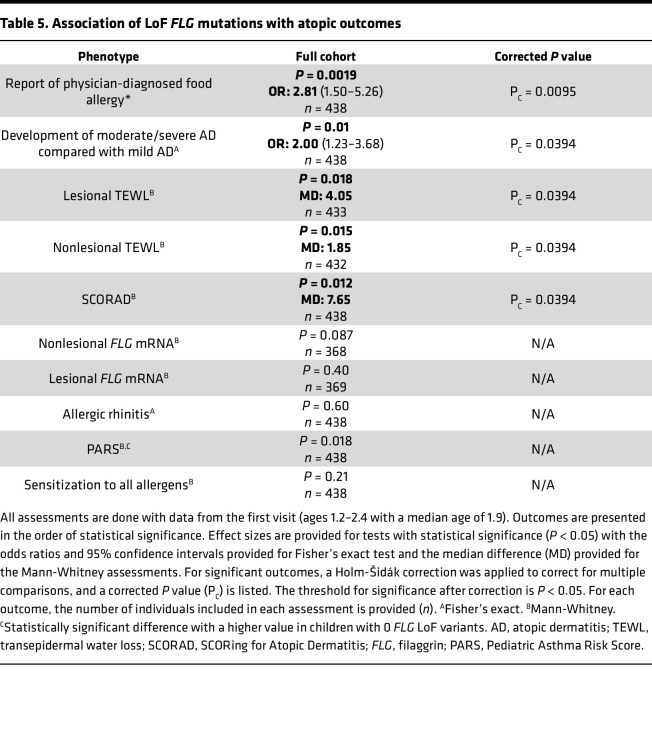
Association of LoF *FLG* mutations with atopic outcomes

## Version 1 04/02/2024

In-Press Preview

## Version 2 05/08/2024

Electronic publication
