# Supplementary material for: Filaggrin loss-of-function variants are associated with atopic dermatitis phenotypes in a diverse, early-life prospective cohort
Source: JCI Insight. 2024 Apr 2;9(9):e178258. doi: 10.1172/jci.insight.178258 (PMC11141906; doi:10.1172/jci.insight.178258)
Supplement: Supplemental data [file jciinsight-9-178258-s102.pdf]

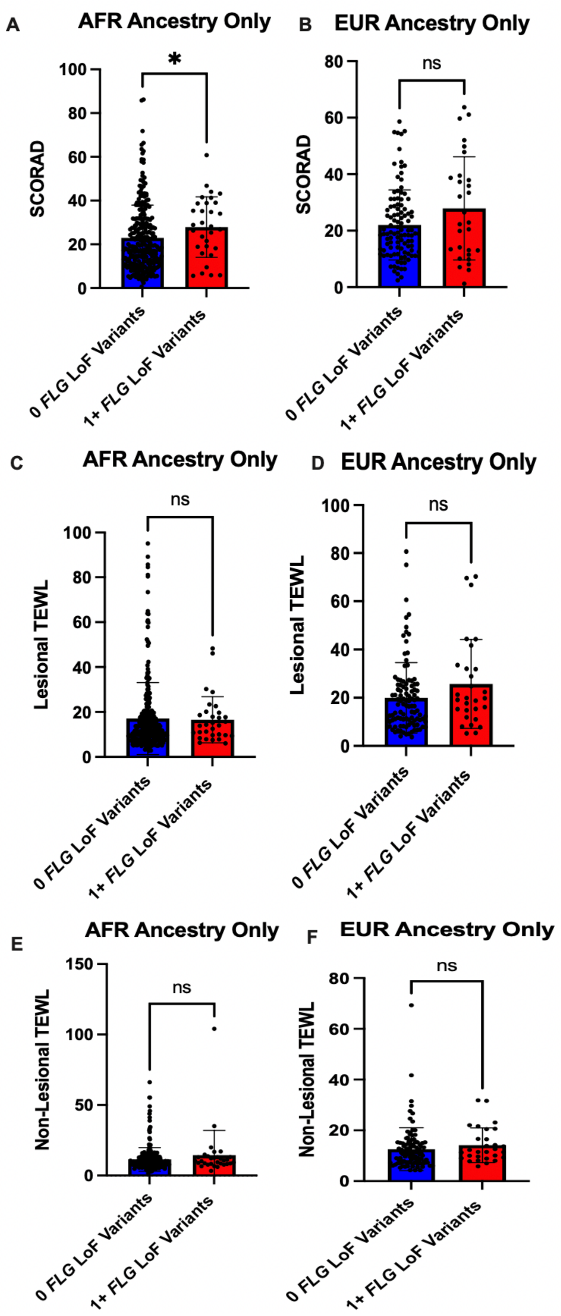

Supplemental Figure 1. *FLG*-dependent risk of early life allergic outcomes in the MPAACH cohort of children with atopic dermatitis broken down by ancestry. The individual level data from B. SCORAD, C. Lesional TEWL, and D. Non-lesional TEWL is presented for subjects characterized by no loss of function *FLG* variants (blue) or one or more loss of function *FLG* variants (red). All assessments were performed with data from the first visit (ages 1.2-2.4 with a median age of 1.9). Mann-Whitney assessments were used to estimate significance. *ns: non-significant; *: P<0.05.*. AD: atopic dermatitis; TEWL: transepidermal water loss; SCORAD: SCOring Atopic Dermatitis; *FLG*: *filaggrin*; PARS: pediatric asthma risk score.

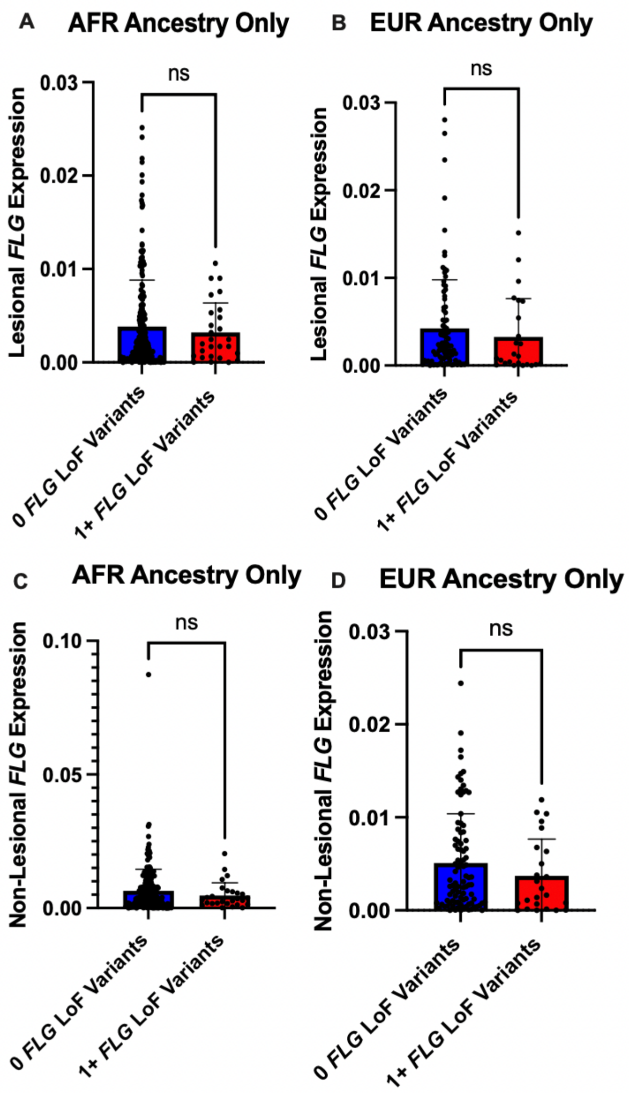

Supplemental Figure 2. *FLG* LoF variants are not associated with *FLG* expression in lesional or non-lesional skin in a cohort of children with AD. All assessments were performed with data from the first visit (ages 1.2-2.4 with a median age of 1.9). Mann-Whitney assessments were used to estimate significance. *ns: non-significant; *: P<0.05.*

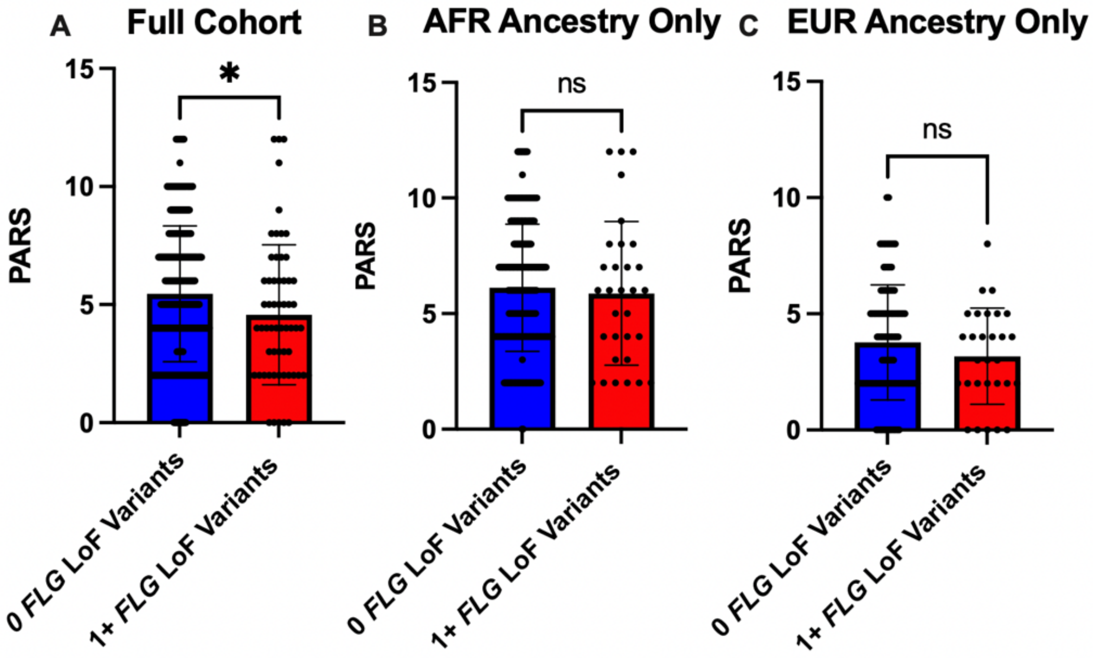

Supplemental Figure 3. *FLG* LoF variants are not associated with PARS in a cohort of children with AD. The individual level data from PARS are presented for subjects characterized by no loss of function *FLG* variants (blue) or one or more loss of function *FLG* variants (red) in the full cohort (A), children of African Ancestry Only (B), and children of European Ancestry only (C). All assessments were performed with data from the first visit (ages 1.2-2.4 with a median age of 1.9). Mann-Whitney Assessments were performed to estimate significance. *ns: non-significant; *: P<0.05. FLG*: filaggrin; PARS: pediatric asthma risk score.

Association	Full Cohort Subjects	Full Cohort Power	AFR Ancestry Subjects	AFR Ancestry Power	EUR Ancestry Subjects	EUR Ancestry Power	Passed Normality Test
**Risk of Food Allergy**	LoF: Y: 18; N: 42<br>Non-LoF: Y: 50; N: 328	87.0%	LoF Y: 6; N: 25<br>Non-LoF: Y: 21; N: 250	55.2%	LoF Y: 12; N: 17<br>Non-LoF: Y: 29; N: 78	12.3%	N/A
**Risk of Moderate-Severe AD**	LoF: Y: 33; N: 27<br>Non-LoF: Y: 128; N: 240	75.2%	LoF: Y: 19; N: 12<br>Non-LoF: Y:100, N: 171	74.7%	LoF: Y: 14; N: 15<br>Non-LoF: Y:38, N: 69	24.3%	N/A
**Lesional TEWL**	LoF Mean + SD Lesional TEWL: 20.99 ± 15.4<br>Non-LoF Mean + SD Lesional TEWL: 17.90 ± 16.68	29.7%	LoF Mean+SD: 16.53±10.27<br>Non-LoF Mean+SD: 17.09±16.04	4.50%	LoF Mean+SD: 25.75±18.47<br>Non-LoF Mean+SD: 19.96±14.60	34.4%	No<br>p<0.0001
**Non-Lesional TEWL**	LoF Mean + SD Non-Lesional TEWL: 14.29 ± 13.37<br>Non-LoF Mean + SD Lesional TEWL: 11.75 ± 8.393	29.7%	LoF Mean+SD: 14.43±17.55<br>Non-LoF Mean+SD: 11.41±8.376	15.5%	LoF Mean+SD: 14.15±6.838<br>Non-LoF Mean+SD: 12.6±8.447	17.4%	No<br>p<0.0001
**SCORAD**	LoF SCORAD Mean+SD: 27.89 ± 15.99<br>Non-LoF: 22.60 ± 14.34	66.0%	LoF SCORAD Mean+SD: 27.90 ± 13.84<br>Non-LoF: 22.95 ± 15.05	46.4%	LoF SCORAD Mean+SD 27.88± 18.27<br>Non-LoF SCORAD Mean+SD: 22.04±12.41	36.8%	No<br>p<0.0001

Supplemental Table 1.Power Calculations and Normality Tests. Results of power calculations for outcomes that had significant associations ($p<0.05$) with *FLG* LoF variant status. For continuous variables, a Shapiro-Wilk test for normality was applied and the p-value is reported. Y: Presence of Outcome; N: Absence of Outcome; AFR: African Ancestry; SCORAD: SCORing for Atopic Dermatitis.
